# DISCOVERING STAFFING ISSUES AND EXPERIMENTING WITH STAFFING LEVELS IN DUTCH NURSING HOMES

**DOI:** 10.1093/geroni/igz038.3258

**Published:** 2019-11-08

**Authors:** Marleen Prins, Janne van Erp, Ceciel Heijkants, Erica de vries, Ramona Backhaus, Sil Aarts, Hilde Verbeek, Astrid van der schot

**Affiliations:** 1 Maastricht University, Faculty of Health Medicine and Life Sciences, CAPHRI, Department of Health Services Research, Maastricht, Netherlands; 2 Maastricht University, Maastricht, Netherlands; 3 Department of Health Services Research, Care and Public Health Research Institute (CAPHRI), Faculty of Health, Medicine and Life Sciences, Maastricht, Netherlands; 4 Netherlands Institute of Mental Health and Addiction (Trimbos-institute), Department on Aging, Utrecht, Netherlands

## Abstract

Background: In 2017, the Dutch National Health Care Institute developed a quality framework describing what high quality nursing home care entails, aimed to improve nursing home care. The current study focuses on evaluating whether Dutch nursing homes comply to the framework, specifically regarding norms about staffing and formation of care teams (skill mix and educational level). Care teams that were experimenting with new staffing levels were monitored to evaluate what changes in staffing occurred and which obstructing or promoting factors they experienced. Methods: Quantitative data about staffing and team characteristics were gathered. Further, qualitative data about motives for wanting to change, the change approach, obstructing and promoting factors and evaluation of changes was collected. Telephone interviews were held at baseline, after 3 months and after 6 months. Thirty-two teams participated in the study. Results: Challenges for making changes in staffing consisted of attracting new care staff, dealing with sickness leave, communication within and between teams, communication with informal carers and combining care for and having attention for well-being of residents in the daily work routine. Additionally, teams wanted to better adjust the skill mix of staff to the needs of residents. Conclusion: For the formation of care teams, there seems to be no ‘one-size fits all’ approach. A quantitative norm that applies to all nursing homes in the Netherlands as described in the quality framework (e.g. a minimum of two care professionals for eight residents during intensive moments of care) is therefore not always the route to high quality care.

